# Antibiotic Resistance in Marine Microbial Communities Proximal to a Florida Sewage Outfall System

**DOI:** 10.3390/antibiotics9030118

**Published:** 2020-03-11

**Authors:** Dale W. Griffin, Kenneth Banks, Kurtis Gregg, Sarah Shedler, Brian K. Walker

**Affiliations:** 1 U.S. Geological Survey, 600 4th Street South, St. Petersburg, FL 33772, USA; 2 Broward County, Environmental Planning and Community Resilience Division, 115 South Andrews Avenue, Room 329-H, Fort Lauderdale, FL 33301, USA; kbanks@broward.org; 3 ERT, Inc., NOAA-Fisheries Service, 400 North Congress Avenue, Suite 270, West Palm Beach, FL 33401, USA; kurtis.gregg@noaa.gov; 4 Biological Oceanography Department, University of South Florida, College of Marine Sciences, 830 1st Street South, St. Petersburg, FL 33701, USA; sshedler@mail.usf.edu; 5 Halmos College of Natural Sciences and Oceanography, Nova Southeastern University, 8000 North Ocean Drive, Dania Beach, FL 33004, USA; walkerb@nova.edu

**Keywords:** marine pollution, antibiotic resistance, sediments, ocean outfall

## Abstract

Water samples were collected at several wastewater treatment plants in southeast Florida, and water and sediment samples were collected along and around one outfall pipe, as well as along several transects extending both north and south of the respective outfall outlet. Two sets of samples were collected to address potential seasonal differences, including 38 in the wet season (June 2018) and 42 in the dry season (March 2019). Samples were screened for the presence/absence of 15 select antibiotic resistance gene targets using the polymerase chain reaction. A contrast between seasons was found, with a higher frequency of detections occurring in the wet season and fewer during the dry season. These data illustrate an anthropogenic influence on offshore microbial genetics and seasonal flux regarding associated health risks to recreational users and the regional ecosystem.

## 1. Introduction

Antibiotic resistance has recently been recognized as an emerging environmental contaminant, and molecular methods that identify these genes in microbial populations have proven to be useful tools in assessing anthropogenic impacts in terrestrial and aquatic environments [1]. One of the primary problems with antibiotics is their widespread use and the slow progress in identifying and developing alternatives such as vaccines and virulence inhibitors [2]. Current estimates for antibiotic resistant infections in the United States of America are 2 million cases annually, of which approximately 23,000 are fatal [3]. Globally, the cost associated with these types of infections is approximately 5.8 trillion USD [4]. In the absence of mitigation, deaths from antibiotic resistant microorganisms may surpass those caused by cancer by 2050, with an estimated economic impact of approximately 100 trillion USD [5].

Relative to a recent increase in understanding the distribution and potential impacts of pharmaceuticals in rivers, authors noted ‘*By contrast, significantly less attention has been paid to understanding releases of pharmaceuticals from sewage and other routes into coastal environments and their potential marine impacts*’ [6]. The presence of antibiotic resistant microorganisms in marine sediments at a polluted site in Tolo Harbour, Hong Kong, was greater than that observed at three other less severely impacted locations [7]. Rivers were identified as a primary source for the discharge of antibiotics into marine environments in a study of Bohai Bay, China, and the highest concentrations of antibiotics coincided with an elevated human presence [8]. The ability of wastewater treatment plants to discharge viable antibiotic resistant bacteria into a marine environment through ocean outfalls was demonstrated in the Gulf of Gdansk, Poland [9]. A study of antibiotic resistance in total and fecal coliforms collected in the vicinity of an ocean outfall pipe in seawater and shellfish samples in the 1970s reported that at least seventy percent of the isolates were resistant to one or more antibiotics [10]. The authors of that study further reported that forty-five percent of the isolates resistant to streptomycin or tetracycline were capable of horizontal gene transfer [10]. Recent research has demonstrated that water disinfection byproducts can increase natural transformation rates [11]. Marine aquaculture studies have highlighted the risk to these types of operations due to their potential influences on the regional prevalence of antibiotic resistant strains of bacteria [12,13].

**Figure 1:**
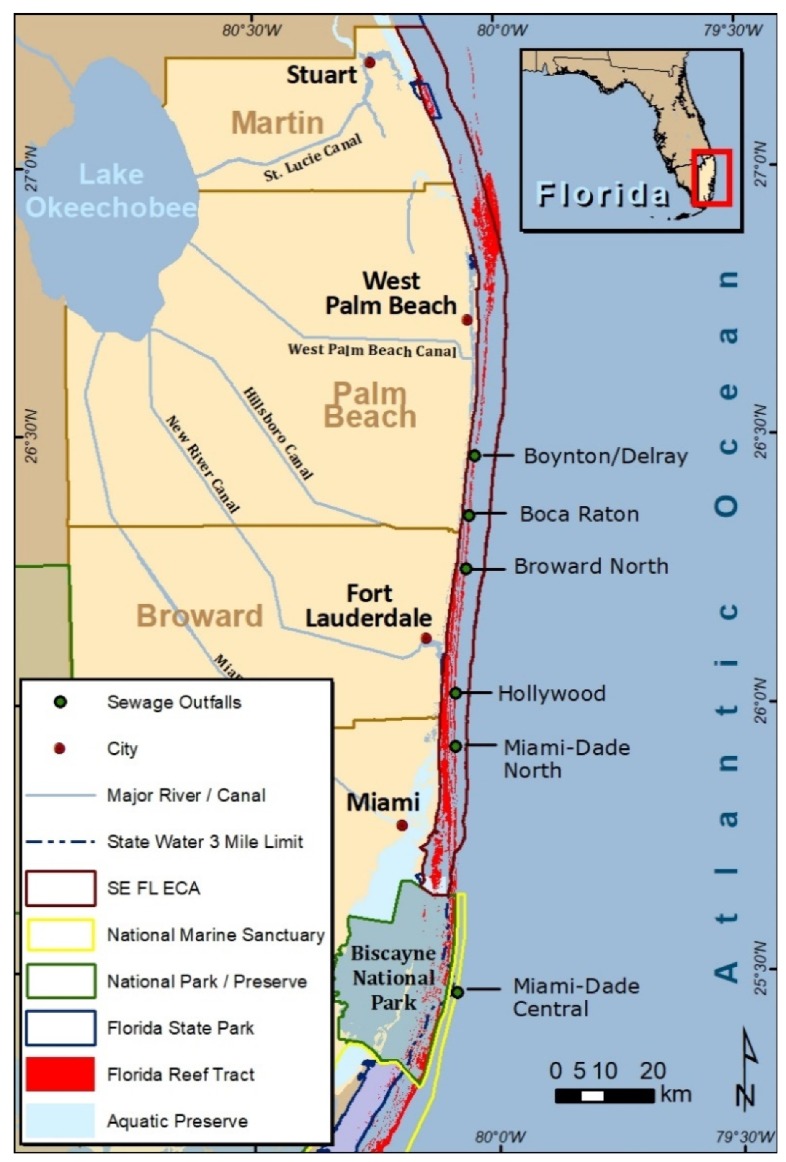
The six ocean outfall systems of southeast Florida.

Analyzing microbial communities for the presence of antibiotics enables researchers to demonstrate at the genetic level, the influences of antibiotic laden sources such as septic systems, shallow injection wells, storm sewer overflows and outfalls on regional ecosystems. As antibiotic resistance can affect pathogen virulence, these sources of antibiotic laden sources of pollution can sustain the presence of these pathogens, which can, in turn, present human recreational and ecosystem health risks. Currently, there are six wastewater ocean outfall systems (known as Boynton-Delray to the north and then progressing to the south, Boca Raton, Broward/North, Hollywood, Miami-Dade North and Miami-Dade Central; Figure 1) in operation in southeast Florida that had a combined flow of 425 million gallons per day (mgd) in 2005 and a projected flow of 474 mgd by the year 2025 [14]. Legislation was passed in 2008 to close these systems by 2025 but this was amended in 2013 to permit occasional use (peak flow) beyond 2025.

The objective of this project was to determine the prevalence of antibiotic resistance genes in bacterial populations in impacted (close to and alongside treated wastewater outfall pipes and within the wastewater stream) and reference (along transects extending away from the outfalls) sediment samples around the Hollywood, Florida wastewater treatment plant’s ocean outfall. Sediment, untreated wastewater, and samples from the outfall pipe outlet boil were analyzed for 15 different antibiotic resistant gene targets via polymerase chain reaction presence/absence assays. A total of 38 samples in the wet season (June 2018) and 42 samples in the dry season (March 2019) were collected by the Florida Department of Environmental Protection, Broward County and National Oceanic and Atmospheric Administration (NOAA) research teams, then shipped to and analyzed at the U.S. Geological Survey (USGS) St. Petersburg Pathogen and Emerging Contaminant Laboratory.

## 2. Material and Methods

### 2.1. Sample Sites and Collection

**Figure 2:**
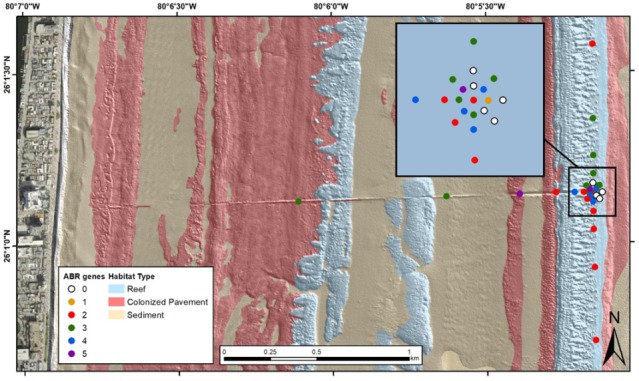
Wet season (June 2018) map of sampling locations and habitat type, Hollywood, Florida, treated wastewater outfall pipe. The number of antibiotic (ABR) genes detected in each sediment sample is listed in the legend. DNA extracts from water collected from the wastewater treatment plant influent and outfall pipe boil were positive for 6 and 3 antibiotic resistance gene targets, respectively.

**Figure 3:**
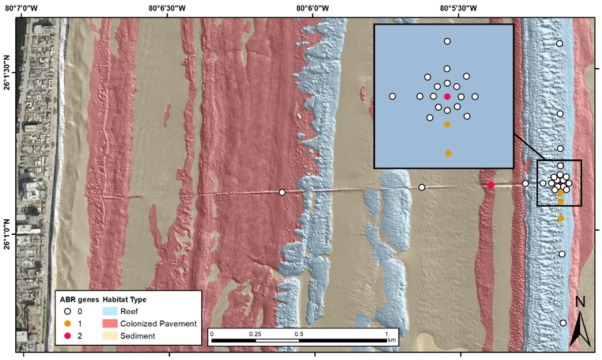
Dry season (March 2019) map of sampling locations and habitat type, Hollywood, Florida treated wastewater outfall pipe. Number of antibiotic (ABR) genes detected in each sediment sample is listed in the legend. DNA extracts from water collected from the wastewater treatment plant influent and outfall pipe boil were positive for 7 and 8 antibiotic resistance gene targets respectively.

Sediment sample sites along the Hollywood, Florida ocean outfall pipe are illustrated in the wet and dry season heatmap figures (Figure 2; Figure 3). Samples included sediment and water column samples collected along transects centered on the exit point of a regional treated wastewater ocean outfall pipe (Hollywood) and from regional wastewater treatment plants (Broward/North, Hollywood and Miami-Dade/North). Like the Hollywood wastewater treatment plant and outfall outlet associated samples (boil and sediment), sample sets collected at the Broward/North and Miami-Dade/North wastewater treatment plants included untreated influent and effluent water samples, an outfall outlet boil sample and a sediment sample collected next to the outfall outlet. Additional data can be found via USGS data release [15].

All sediment samples were collected by scuba divers who navigated to the sample site by descending an anchored center-point buoy line (centroid) and attaching a measuring tape to the anchor. Divers collected one sediment sample at the centroid of the array near the anchor (beneath the exit point of the outfall pipe) and then navigated to the cardinal and ordinal directions (N, NW, W, SW, S, SE, E, NE), by dive scooters or by self-propulsion, to collect samples at 25 m and 50 m from the centroid. Samples at 100, 200, 400 and 800 m north and south of the centroid were collected on single dives at those intervals. Samples collected at 100, 200, and 400 m west (shoreward) of the centroid were collected by one diver team using scooters to move shoreward from site to site along the pipeline, with anchored floats deployed from the boat marking the collection location. Samples at 800 and 1600 m from the centroid were collected on individual dives at those sites. Sediment samples were collected by divers using a 50 mL centrifuge tube to scoop sediment from the seabed. Divers wore single-use nitrile gloves to prevent cross-contamination of sediment samples. Nitrile gloves were changed between samples (underwater) if more than one sample was collected on a dive. Outfall water samples were collected by opening an empty sterile 50 mL centrifuge tube in the plume (identified by a change in water color and higher velocity than surrounding water) at the mouth of the pipe. Wastewater treatment plant effluent water samples from the three wastewater treatment plants were collected while wearing nitrile gloves. Influent and effluent samples were collected directly from spigots using sterile 50 mL centrifuge tubes. These effluent samples were then placed in plastic Ziploc bags and kept on ice during transport to the Florida Department of Environmental Protection (FDEP) Coral Reef Conservation Program (CRCP) office, and then placed in a freezer with the field samples.

### 2.2. Sample Storage and Shipping

Field sediment and water samples representing the wet (11–20 June 2018) and dry (4–11 March 2019) seasons were brought to the surface, where they were kept on ice on the boat and during transport to the FDEP CRCP office in Miami, FL, and then placed in a cryogenic freezer (wet season) or refrigerator (dry season). Effluent samples were also stored in a cryogenic freezer. Wet season samples were shipped overnight on dry ice to the USGS lab in St. Petersburg, FL (received 24 July 2018) once the interagency agreements were completed. Dry season samples were shipped overnight once the last of the samples had been collected (received 12 March 2019). All samples were stored at −20 °C upon receipt by the USGS until processed for DNA extraction.

### 2.3. Antibiotic Resistance Presence/Absence Polymerase Chain Reaction Assays

DNA was extracted from samples as previously published [1]. In short, ~250 µl of water samples and 0.25 g of sediment were utilized to extract DNA using the DNeasy PowerSoil Kit (Qiagen, Hilden, Germany). The only modification to the Kit protocol instructions was the elution with Qiagen AE buffer (100µl, limits DNA degradation during frozen storage vs the traditional MoBio PowerSoil Kit eluent) instead of the Kit eluent. An amount of 2 µl microliters of this purified DNA was were utilized for templates in duplicate reactions for each gene target. Applied Biosystems TaqMan Fast Universal Master Mix and TaqMan Exogenous Internal Positive Control reagents were utilized in all 20µl reactions. Primer/probe sequences (Table 1), probe labels, amplification profiles, PCR plate layouts and master mix conditions were as previously published and are available as previously published and via USGS data release [1,15]. TaqMan exogenous internal positive control reagents were used in duplicate reactions for each target gene. Target genes are listed in Table 2 and included *aadA2, ampC, blaPSE, blaSHV, ermB, floR, mecA, tetB, tetG, tetL, tetM, tetO, tetQ, tetW* and *vanA*. Positive controls were gene target sequences synthesized with ~15–25 base pair extensions beyond the 5′ and 3′ ends of the primer binding sequences. The controls were synthesized by Integrated DNA Technologies (gBlock double-stranded DNA fragments).

## 3. Results

### 3.1. Wet Season

The southeast Florida wet season sample set was composed of six wastewater samples collected at three different wastewater treatment plants (Broward/North, Hollywood, and Miami-Dade/North), their respective outfalls, and 32 sediment samples collected at those outfalls, and in an array and along transects centered on the Hollywood outfall. Nine of the antibiotic resistance genes were detected in the sample set. The most prevalent antibiotic resistance genes detected in the samples were *tetW* and *aadA2* at 68.4% and 60.5%, respectively. Seven (*tetB, tetW, ampC, vanA, ermB, mecA* and *tetQ*) of the fifteen antibiotic resistance genes were detected in the wastewater treatment plant samples/outlet boil samples and six (*tetO, tetW, ampC, vanA, mecA* and *aadA2*) were detected in the offshore sediment samples. *tetB, ermB* and *tetQ* were only detected in the wastewater samples and *tetO* and *aadA2* were only detected in the sediment samples. *tetL, tetM, blaSHV, blaPSE, floR* and *tetG* were not detected in any of these samples. Table 3 lists the number and identification of antibiotic resistance gene detections per sample type. Figure 2 illustrates the occurrence of the different numbers of antibiotic resistance genes detected at each site in the offshore environment around the Hollywood, Florida, outfall pipe. Tabled site-specific sample data are available via USGS data release [15].

### 3.2. Dry Season

The dry season sample set was composed of 10 wastewater samples collected at three different wastewater treatment plants (Broward/North, Hollywood, and Miami-Dade/North), their respective outfalls, and 32 sediment samples collected at those outfalls, and in an array and along transects centered on the Hollywood outfall. Ten of the antibiotic resistance genes were detected in the sample set. The most prevalent antibiotic resistance genes detected in the samples were *ermB* and *tetW* at 35.7% and 31.0%, respectively. Ten (*tetB, tetM, tetO, tetW, ampC, vanA, ermB, mecA, blaSHV* and *tetQ*) of the fifteen antibiotic resistance genes were detected in the wastewater plant/outfall boil samples and four (*tetW, ampC, vanA* and *ermB*) were detected in the sediment samples. *tetB, tetM, tetO, mecA, blaSHV* and *tetQ* were only detected in the wastewater associated samples and all of the four antibiotic resistance genes found in the sediment samples were also detected in the wastewater samples. *tetL, blaPSE, floR* and *tetG* were not detected in any of these samples. Figure 3 illustrates the occurrence of the different numbers of antibiotic resistance genes detected at each site in the offshore environment around the Hollywood, Florida outfall pipe. Tabled site-specific data are available via USGS data release [15].

## 4. Conclusion

The southeast Florida data illustrates that antibiotic resistance genes are readily detectable in the wastewater stream and in sediments close to and alongside the outfall outlet and along the outfall pipe. The shoreward run of the outfall pipe lies in a trench so it may act as a sink for sediment at the pipe outlet that are resuspended during storm conditions and advected shoreward. The wet season data set shows widespread occurrence of multiple antibiotic resistance genes, with the highest instances occurring in the wastewater stream, alongside the Hollywood outfall pipe, and in close proximity to the outfall outlet. The dry season results show a concentrated occurrence in the wastewater stream but less offshore occurrence relative to the wet season data. The offshore positive samples are associated along the outfall pipe, the outfall outlet, and along the southern transit. The prevalent data between the seasons are interesting, with the wet season showing a lower prevalence in the wastewater stream and higher prevalence in the sediment samples and the opposite trend occurring in the dry season. This observed trend of a greater prevalence of these antibiotic resistance genes in microbial sediment communities in close proximity to the outfall system may be due to a seasonal temperature flux. The wet season occurs during the warmer months of the year, and these higher temperatures likely result in elevated metabolic rates and genetic exchange within these communities. Seasonal differences in antibiotic usage may also influence the presence or prevalence of resistance genes in these environments. Outfall discharge rates could also contribute to exposure and genetic response in these microbial communities, but in this case, similar outfall discharge rates between these two sample seasons were noted at 11.9 mgd for June 2018 and 12.1 mgd for March 2019 (data courtesy of the City of Hollywood).

Sewage associated wastewater is well known to carry numerous antibiotics due to public health use. Microbial communities under the influence or stress of antibiotic laden wastewater will acquire resistance. Microbial communities are known to share resistant genes within and across genera when there is an exposure source, even at a heightened metabolic cost [19,20]. The southeast Florida ocean outfall sediment and water sample data illustrate that type of microbial response. The assay results indicate that there is a heightened prevalence of these genes in the wet season that may be due to factors such as seasonal water temperatures, increased seasonal wastewater discharge flows, seasonal antibiotic usage and coastal flow dynamics. Members in these coastal microbial communities may present risks to recreational water use and to the ecosystem itself. In regard to these southeast Florida outfall systems and their permitted peak flow use beyond 2025, these peak flow events usually occur during Florida’s wet season, which typically starts in June of each year. The data observed in this study should be considered in future seasonal scale risk analyses that are conducted in regard to ocean outfalls that are in close proximity to sensitive marine ecosystems and waters utilized for recreational use.

## Figures and Tables

**Table 1:** Antibiotic resistance gene primer and probe set sequences.

Gene Target	Upstream Primer	Downstream Primer	Probe	Reference
*aadA2*	CAGCCAYGATCGACATTGATCT	CCAAGGCAACGCTATGTTCTC	CTGCTTACAAAAGC	[16]
*ampC*	GGGAATGCTGGATGCACAA	CATGACCCAGTTCGCCATATC	CCTATGGCGTGAAAACCAACGTGCA	[17]
*bla*_PSE_	GATTTGGTGCTCGGAGTATT	CATTGAAGCCTGTGTTTGAG	CTTGATGCTCACTCCA	[16]
*bla*_SHV_	AACAGCTGGAGCGAAAGATCCA	TGTTTTTCGCTGACCGGCGAG	TCCACCAGATCCTGCTGGCGATAG	[18]
*ermB*	GGATTCTACAAGCGTACCTTGGA	GCTGGCAGCTTAAGCAATTGCT	CACTAGGGTTGCTCTTGCACACTCAAGTC	[18]
*floR*	GGCAGGCGATATTCATTACT	CGAGAAGAAGACGAAGAAGG	CTAAAGCCGACAGTGTA	[16]
*mecA*	CATTGATCGCAACGTTCAATTTAAT	TGGTCTTTCTGCATTCCTGGA	CTATGATCCCAATCTAACTTCCACATACC	[18]
*tetB*	ACACTCAGTATTCCAAGCCTTTG	GATAGACATCACTCCCTGTAATGC	AAAGCGATCCCACCACCAGCCAAT	[17]
*tetG*	CGGTACTTCTGGCTTCTCTT	GAATCGGCAATGGTTGAG	CAGGAGCCGCAGTCGATTACACG	[16]
*tetL*	GGTTTTGAACGTCTCATTACCTGAT	CCAATGGAAAAGGTTAACATAAAGG	CCACCTGCGAGTACAAACTGGGTGAAC	[17]
*tetM*	GGTTTCTCTTGGATACTTAAATCAATCR	CCAACCATAYAATCCTTGTTCRC	ATGCAGTTATGGARGGGATACGCTATGGY	[17]
*tetO*	AAGAAAACAGGAGATTCCAAAACG	CGAGTCCCCAGATTGTTTTTAGC	ACGTTATTTCCCGTTTATCACGG	[17]
*tetQ*	AGGTGCTGAACCTTGTTTGATTC	GGCCGGACGGAGGATTT	TCGCATCAGCATCCCGCTC	[17]
*tetW*	GCAGAGCGTGGTTCAGTCT	GACACCGTCTGCTTGATGATAAT	TTCGGGATAAGCTCTCCGCCGA	[17]
*vanA*	CTGTGAGGTCGGTTGTGCG	TTTGGTCCACCTCGCCA	CAACTAACGCGGCACTGTTTCCCAAT	[17]

**Table 2:** Antibiotic resistance genes and the antibiotics to which they provide resistance.

Antibiotic Resistance Gene	Examples of Affected Antibiotics/Drugs
*aadA2*	Streptomycin, spectinomycin
*ampC*	beta-lactams (ampicillin, penicillin, etc.)
*blaPSE*	beta-lactams
*blaSHV*	beta-lactams
*ermB*	Macrolides (erythromycin, etc.), lincosamides (lincomycin, etc.), streptogramins (synercid, etc.)
*floR*	Florfenicol, chloramphenicol
*mecA*	Methicillin
*tetB*	Tetracycline
*tetG*	Tetracycline
*tetL*	Tetracycline
*tetM*	Tetracycline
*tetO*	Tetracycline
*tetQ*	Tetracycline
*tetW*	Tetracycline
*vanA*	Vancomycin

**Table 3:** Number and identification of antibiotic resistance gene detections per sample type.

Sample Type	Number of Samples	Total Number of Antibiotic Resistance Gene Detections	Antibiotic Resistance Genes Detected
Wet Season samples			
Wastewater treatment plant	3	11	*tetB, tetQ, ampC, ermB, vanA*
Outfall boil	3	6	*tetB, tetQ, tetW, ermB*
Sediment	32	77	*tetO, tetQ, tetW, ampC, ermB, vanA, mecA, aadA2*
Dry Season samples			
Wastewater treatment plant	7	46	*tetB, tetM, tetO, tetQ, tetW, ampC, ermB, vanA, mecA, blaSHV*
Outfall boil	3	22	*tetB, tetO, tetQ, tetW, ampC, ermB, mecA, blaSHV*
Sediment	32	11	*tetW, ampC, ermB, vanA*
